# Dietary intakes and nutritional status of children with sickle cell disease at the Princess Marie Louise Hospital, Accra – a survey

**DOI:** 10.1186/s40795-018-0241-z

**Published:** 2018-08-23

**Authors:** Isaac Boadu, Agartha Ohemeng, Lorna Awo Renner

**Affiliations:** 1 Department of Nutrition and Food Science, University of Ghana, Accra, Ghana; 2 Department of Child Health, Korle-Bu Teaching Hospital, Accra, Ghana

**Keywords:** Sickle cell disease, Nutritional status, Dietary intake, Anaemia, Nutritional management

## Abstract

**Background:**

Sickle cell disease (SCD) is a chronic genetic blood disorder common among people of African descent, which places nutritional burden among affected individuals. The aim of the study was to determine the dietary intake and nutritional status of children with SCD.

**Method:**

This was a cross sectional study that involved 120 children with SCD aged 3–12 years at the paediatric outpatient department of Princess Marie Louise Hospital (PML) in Accra. A semi-structured questionnaire was used to take information on participant’s demographic characteristics and clinical data were obtained from their medical records. Dietary data were obtained using 24-h dietary recall and food frequency questionnaire. Height and weight were measured for participants and their haemoglobin levels were determined to assess their anaemia status.

**Results:**

Intakes of participants were below recommendations for most nutrients, particularly for calcium and antioxidant vitamins (vitamin C and E). Adequacy of nutrient intake declined with age and children with genotype HbSS had a lower tendency to meet dietary recommendations (aOR = 0.45, 95% CI: 0.18, 1.10; *p* = 0.084). More than a third of the study children (38%) were malnourished, with older children having a tendency to be malnourished compared to the younger ones. Children with the SS genotype were more likely to be stunted (aOR = 3.48, 95% CI: 1.10, 11.01), compared to other genotypes.

**Conclusion:**

Malnutrition is prevalent among children with SCD and hence there is the need to develop comprehensive management coupling nutritional therapy to medical care to improve their nutritional status. Nutritional management should focus much on calcium-rich foods and antioxidants nutrients particularly vitamin C and E to reduce rapid erythrocyte haemolysis and chronic anaemia.

## Background

Sickle cell disease (SCD) is recognized as one of the common chronic genetic blood disorders among humans with variable clinical manifestations. It can be referred to as one of the underappreciated and least addressed contributors to childhood morbidity and mortality globally. The disease occurs as a result of a point mutation in the β-globin chain of haemoglobin molecule; where a non-polar amino acid, valine, is substituted for a polar amino acid, glutamic acid [1]. In a low oxygen setting, this single amino acid substitution leads to polymerization of haemoglobin molecules resulting in sickle shaped red blood cells. The main consequences resulting from this abnormality are vaso-occlusive events and increased haemolysis. Vaso-occlusive events may lead to tissue, bone, and organ damage while chronic haemolysis may lead to anaemia with a base line haemoglobin level as low as 6.0 g/dl [2]. Comorbidities such as pain, stroke, and anorexia that are associated with SCD have been shown to decrease dietary intake leading to impaired growth, poor nutritional status, and delayed skeletal and sexual maturation [3].

Majority of children born with SCD occur in the developing world, with an estimated 200,000 annual HbSS births in sub-Saharan Africa [4]. In Ghana, recent studies indicate that 2% (14000) of annual newborns are affected by SCD and 25% of the Ghanaian population are carriers of the sickle cell gene. One in three Ghanaians have the haemoglobin S and/or C gene [5]. Information on SCD has mainly been reported among US population, India and Jamaica, with less information in Africa where the gene is highly predominant. Although some studies [6, 7] have reported on nutritional status of children with SCD in parts of Africa, there has been paucity of studies designed to identify the dietary intake of children with SCD in Ghana. A study in Ghana [8] reported on high prevalence of malnutrition (61.3%) among children with SCD but did not consider their dietary intake. Emphasis on dietary intakes of SCD children have thus, been inadequately documented in Ghana. The purpose of this study was to assess the dietary intake and nutritional status of children aged 3–12 years with SCD.

## Methods

This was a hospital-based cross sectional study carried out at the paediatric outpatient department of Princess Marie Louise Hospital (PML) in Accra, Ghana. The hospital provides paediatric services to patients with all forms of medical conditions including SCD. The sickle cell clinic is held once a week. All SCD patients are scheduled for regular appointments every three or four months depending on child’s condition. On average, about 20 patients are seen at the clinic every week including newly diagnosed cases.

The study participants included children aged 3–12 years diagnosed with SCD who were attending outpatient sickle cell clinic at PML. Recruitment was done as the children awaited medical care. Children were recruited if they were in steady state (not having a crisis) and free from other chronic medical conditions such as HIV, tuberculosis, etc. They were excluded if they were suffering from conditions known to affect dietary intake such as cleft palate and sores in the mouth or throat. The study protocol was approved by the Ethical Committee of the College of Basic and Applied Sciences (ECBAS), University of Ghana, and Ghana Health Service (GHS) Ethical Review Committee (ECBAS 008/15–16 and GHS-ERC 05/08/15 respectively). Informed written consent was obtained from the parent or guardian, and oral assent was obtained from each child.

A semi-structured questionnaire was used to take information on participant’s demographic characteristics, and clinical data (such as genotype of children, past admissions and transfusions) were obtained from their medical records. Dietary data were obtained by a single 24-h dietary recall and food frequency questionnaire [9]. Children with the help of their caregivers were asked to recall all foods and beverages consumed during the preceding 24-h period. Household food models were used to aid participants to estimate quantity of foods consumed. Dietary intakes were converted into energy and nutrients using Food Composition Tables based on Ghanaian foods [10].

Height (to the nearest cm) and weight (to the nearest kg) were measured and recorded for each child following standard techniques [11].

A single drop of blood from a finger prick was placed on a test strip in a haemoglobinometer (URIT-12) and the haemoglobin level (g/dl) recorded for each participant.

### Data analyses

Data entry and analyses were conducted using SPSS version 20 (SPSS Inc., Chicago, Ill., USA). Z-scores for height-for-age (HAZ), weight-for-age (WAZ), and body-mass-index for age (BMIZ) were calculated for children using the WHO anthro software (V1.04). Stunting, underweight, and wasting were defined respectively as, a height-for-age z-score (HAZ), a weight-for-age z-score (WAZ), and a weight-for-height z-score (WHZ) less than two standard deviations below the mean values for these scores based on international growth standard data. Of the 120 children, WAZ was computed for 115 children since the WHO anthroplus (V1.04) does not compute WAZ for a child whose age is above 120 completed months. Weight for height Z-score (WHZ) was computed for children less than 5 years (*n* = 44), while BMI-for-age Z-score was computed for children 5 years and above (*n* = 76). A child was further classified as malnourished if he or she had any of the nutritional deficits (stunted, wasted, thin, or underweight), and well-nourished if none of these were detected. Study children were classified as having anaemia based on age-specific haemoglobin cut-offs [12].

Nutrient intakes were presented in means and standard deviations for specific age groups (3, 4–8, and 9–12 years) and also expressed as percentage of the Recommended Daily Allowances (RDA). RDAs were used instead of the Estimated Average Requirement because of the suggested increased nutrient requirements of SCD patients in the literature [13, 14] to account for the increased demand of nutrients. An evaluation of the number of nutrients assessed for which each child had adequate intake was done. For energy and each of the selected nutrients, a score of one (1) was assigned for meeting recommendation and zero (0) for not meeting requirement. These were then summed up to give a summary score for adequacy. From this summary score, a categorical variable was created based on meeting at least five (includes energy) of the nutrients assessed. Data were presented as means with standard errors of mean or frequencies and proportions.

Multiple logistic regression was used to determine demographic and clinical factors associated with dietary intakes (meeting requirements) and nutritional status (stunting and underweight). Wasting and thinness were not included as dependent variables due to the small number of children within these categories. All analyses were considered statistically significant at *p* < 0.05.

## Results

The study involved 120 SCD children, of which 53.3% were males. The mean age of the children was 5.9 ± 2.2 years. Genotypes recorded were SS (71.7%), SC (27.5%) and SF (0.8%) and more than half (50.8%) of the participants were diagnosed with SCD between the first and second year of life (Table 1). Almost half (42.5%) of the participants had previously been on admission and the main reasons for admissions were vaso-occlusive episodes (92.2%) and anaemia (7.8%).

**Table 1:** Clinical information of participants

Variable	n (%)
Sickle cell type	
SS	86 (71.7)
SC	33 (27.5)
SF	1 (0.8)
Age of diagnosis (yrs)	
< 1	12 (10.0)
1–2	61 (50.8)
3–4	40 (33.3)
≥ 5	7 (5.8)
Admission past one year	
Yes	51 (42.5)
No	69 (57.5)
Reason for Admission	
Vaso-occlusive crisis	47 (92.2)
Anaemia	4 (7.8)

**Fig. 1:**
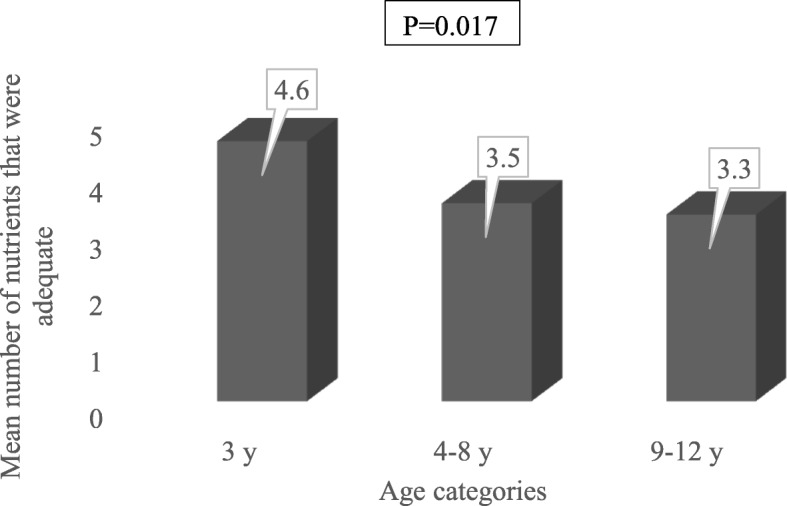
Mean number of nutrients for which study children consumed adequate amounts. On the average, children aged three years met requirement for almost five nutrients that were assessed, but the older age groups (4-8y, 9-12y) met less than four

The mean energy and protein intakes of study children were 1347.9 ± 55.3 Kcal and 56.2 ± 3.5 g, respectively (Table 2). When the intakes of selected nutrients were compared to the RDA, it was observed that almost all the study children (95.8%) met protein requirement (Table 2), but no child met requirements for vitamin E and calcium. Additionally, less than half of the participants met estimated requirements for the following: vitamin B12 (46.7%), vitamins A and C (45% each), zinc (30.8%), energy (28.3%), folate (9.2%), and magnesium (1.7%). Meeting nutrient requirements significantly declined with increase in age (Fig. 1). On the average, children aged three years met almost five nutrients that were assessed, but the older age groups met less than four. More than half of the children aged three (53.3%) met recommendations for energy and at least four of the ten nutrients that were investigated. On the other hand, less than a quarter of the children aged 4–8 years (22.2%) and 9–12 years (20%) met recommendations for at least five nutrients (includes energy). Adjusting for age and sex, children with genotype HbSS had a lower tendency to meet dietary recommendations (aOR = 0.45, 95% CI: 0.18, 1.10; *p* = 0.084) when compared to other genotypes combined (HbSC and HbSF).

**Table 2:** Dietary intakes of children with sickle cell disease, based on a single 24-h recall

Nutrient	Daily Mean intake^a^ *n* = 120	Proportion of children who met recommended values^b^		
		3 years (*n* = 15)	4–8 years (*n* = 90)	9–12 years (n = 15)
Energy (Kcal)	1347.9 ± 55.3	8 (53.3)	14 (15.6)	12 (80.0)
Protein (g/kg)	56.2 ± 3.5	15 (100.0)	87 (96.7)	13 (86.7)
Iron (mg)	11.3 ± 0.6	11 (73.3)	47 (52.2)	11 (73.3)
Zinc (mg)	4.3 ± 0.2	7 (46.7)	28 (31.1)	2 (13.3)
Calcium (mg)	167.7 ± 9.0	0 (0.0)	0 (0.0)	0 (0.0)
Magnesium (mg)	63.5 ± 2.8	0 (0.0)	2 (2.2)	0 (0.0)
Vitamin A (μg RE)	543.2 ± 53.6	9 (60.0)	41 (45.6)	4 (26.7)
Folate (μg)	116.1 ± 6.3	2 (13.3)	9 (10.0)	0 (0.0)
Vitamin B12 (μg)	1.1 ± 0.1	10 (66.7)	41 (45.6)	5 (33.3)
Vitamin C (mg)	74.7 ± 15.9	7 (46.7)	45 (50.0)	2 (13.3)
Vitamin E (mg)	1.1 ± 0.3	0 (0.0)	0 (0.0)	0 (0.0)

^a^Data expressed as mean ± standard error of mean

^b^Intakes based on a single 24-h dietary recall were compared to age-specific Recommended Dietary Allowance (RDA). Values presented as number (%)

### Nutritional status of participants

**Fig. 2:**
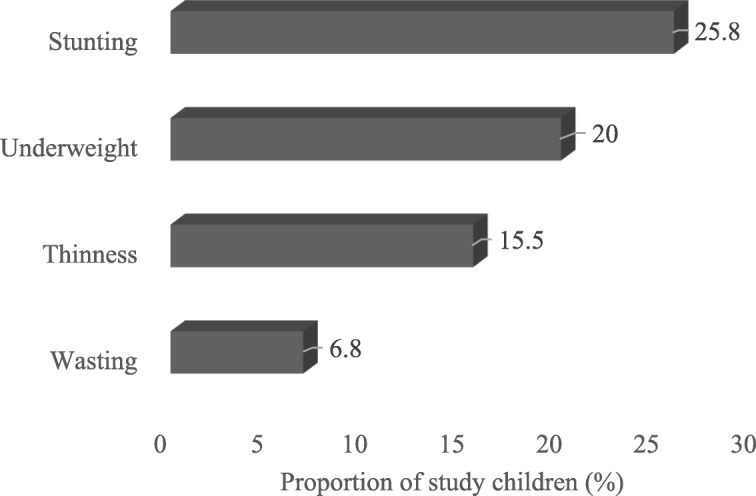
Anthropometric indices of the children. The prevalence of stunting, underweight, wasting (children < 5 yrs) and thinness (children > 5 yrs) among the children were 25.8, 20.0, 6.8 and 15.8% respectively

The mean height-for-age Z-score (HAZ) for all the children was − 0.86 ± 1.4 while the mean weight-for-age (WAZ) and BMI-for-age were − 0.84 ± 1.1 and − 0.74 ± 1.1, respectively. The prevalence of stunting, underweight, wasting (children < 5 yrs) and thinness (children > 5 yrs) among the children were 25.8, 20.0, 6.8 and 15.8% respectively (Fig. 2). Anthropometric indices did not differ significantly between boys and girls (*p* > 0.05). Based on the presence of at least one malnutrition indicator assessed in the study (stunted, wasted, thin, or underweight), 38% of the children were classified as malnourished. After controlling for demographic factors, children with genotype HbSS were 3.5 times more likely to be stunted (Table 3) compared to the other genotypes (SC, SF). In addition, children older than 5 years of age had a higher tendency (*p* = 0.065, 0.098) to be underweight compared to the younger children. Almost all the children (98.3%) were anaemic, with mean haemoglobin of 7.8 ± 1.4 g/dl. Most of them (94.1%) were suffering from moderate to severe forms of anaemia.

**Table 3:** Relationships between genotype and nutritional status, based on a multiple logistic regression

Variables	Dietary adequacy^a^		Stunting		Underweight	
	aOR	95% CI	aOR	95% CI	aOR	95% CI
Age (years)						
< 5	1		1		1	
5–8	0.41	0.09, 1.76	1.38	0.38, 5.06	3.29	0.71, 15.29
9–12	1.00	0.24, 4.23	1.87	0.52, 6.74	2.69	0.65, 11.20
Sex						
Male	1		1		1	
Female	0.52	0.22, 1.26	1.27	0.53, 3.03	0.68	0.25, 1.82
Genotype						
Others^b^	1		1		1	
SS	0.45^*^	0.18, 1.10	3.48^**^	1.10, 11.01	1.54	0.51, 4.66

^a^Meeting the RDA of at least five nutrients assessed, including energy

^b^This includes genotypes SC and SF

^*^p < 0.10, **p < 0.05

## Discussion

SCD does not only affect the rheological property of RBCs but places a nutritional burden on affected individuals. In this present study, there was low intake of nutrients such as folate, magnesium, calcium, vitamin A, and the antioxidant nutrients, vitamin C and E. The low folate intake is consistent with two previous studies [15, 16] where the authors reported that more than half of SCD children had inadequate intake of folate. The low folate intakes support the basis for supplementation to compensate for the chronically increased haemolysis and haematopoiesis among patients with SCD. The study children were routinely prescribed 5 mg of folic acid, as described elsewhere, to address chronically increased haemolysis and haematopoiesis [17, 18]. This study however, did not assess the folate status of the participants and there is therefore, no information on the effectiveness of the supplementation in improving folate status in Ghana.

In sickle cell patients, optimal intake of vitamin A may be important to improve iron absorption, enhance erythropoiesis and immunity, increase the mobilisation of iron from tissue stores and possibly affect red blood cell differentiation [19]. The low vitamin A intake observed in the current study is in accordance with findings reported by Schall et al. [20], but contrast to the findings of an earlier report [21]. Consumption of fruits and green leafy vegetables rich in vitamin A among the children was low and this could account for the observed difference.

Protecting and maintaining red blood cell membranes from free radical damage is pertinent in the management of SCD. Experimentally, the antioxidant nutrients vitamin C and E have been shown to exhibit inhibitory role in red blood polymerisation and have been suggested that a combination of antioxidants would be effective in reducing the incidence and severity of sickle cell crisis [22, 23]. The low intakes of these antioxidants, vitamin E and C is consistent with prior research [24] which indicates that sickle cell children in United States had low intake of vitamin E and other nutrients. These low intakes might have contributed to the high number of SCD related admissions (vaso-cclusive crisis and anaemia) observed in this study.

Calcium is well-recognised for its role in strengthening the bones for erythropoiesis. This makes the mineral an important nutrient in sickle cell patients. Our finding that none of the children studied met the recommended requirement for this macromineral is in line with the findings of Meeuwes et al. [25] among Brazilian sickle cell children and young adults. Other investigators [26–29] have also reported lower intake of calcium among sickle cell patients. It is difficult to meet calcium requirements from other food sources without liberal consumption of dairy products [30]. Although 38 and 18.3% of the children ate from milk and milk products and dark green leafy vegetables food groups which are rich in calcium (data not shown), they were not adequate to meet the recommended daily requirement. The small quantities of milk consumed are characteristics in most Ghanaian communities particularly among those in the Southern part of Ghana where the study was conducted.

Assessment of the daily intake among American children with SCD, showed a declining trend in intake with increasing age, especially during adolescence [24]. This current study confirms this finding in that meeting nutrient requirement declined with age. This may be due to the fact that as age increases, dietary intake might have remained the same such that it may not be adequate to meet the increasing nutrient requirements.

Malnutrition is common among children with sickle cell disease as indicated by delay in sexual maturation, deficits in weight and height, [3] and low bone mineral density [31]. An earlier study in Ghana by Osei-Yeboah et al. [8] reported high prevalence of malnutrition (61.3%) among sickle cell children attending routine check-up at Korle-Bu Teaching hospital. The authors reported significantly more stunted and underweight sickle cell children compared to their normal (HbAA) counterparts. This is similar to our findings, 38.0% of the participants were malnourished, 25.8% were stunted and 20.0% were underweight. Sickle cell anaemia (SS) has been associated with stunting and wasting in a cohort of Tanzania children [6] similar to our finding; SS genotype was significantly associated with stunting. The greater percentage of sickled erythrocytes found in the severe form of SCD, HbSS genotype; result in exacerbated complications including undernutrition, increased nutrient requirements, and low dietary intake. Our study showed that SS children had a lower tendency to meet nutrient requirements. These may explain this finding.

Almost all the children were anaemic. The root cause of anaemia in sickle cell children is from chronic haemolysis due to loss of erythrocyte membrane integrity attributed to deficiency of antioxidant nutrients, folate or iron deficiency and depression of erythropoiesis (aplastic crisis) [32]. Folate is required for the production of red blood cells while vitamin C and E serve as potent inhibitors of sickle cell haemoglobin polymerisation [22]. The low intake of folate and antioxidants vitamins (E and C) by the children may have contributed to the high prevalence of anaemia observed in this study. Although more than half of the children met nutrient requirement of iron, the anaemia prevalence could also be due to the low intake of vitamin C to aid in iron absorption. The current study evaluated many micronutrients intake and the findings add to the body of knowledge of dietary intake and nutritional status of children with SCD. However, the use of a single 24 h dietary recall to ascertain nutrient intakes may not be a representative of usual intake due to day to day variation of food intake and may have led to over estimation of children with lower nutrient intakes. Additionally, comparison with controls would have given a better inference of nutrient intakes and nutritional status of this cohort of children.

## Conclusion

This study shows that children with SCD have low intake of energy and micronutrients particularly calcium and the antioxidant nutrients, vitamin C and E. Age trend showed that meeting nutrient requirements declined with increase in age and children with the genotype SS had a lower tendency to meet recommended daily allowances for nutrients. The prevalence of malnutrition was 38%, with the prevalence of stunting, underweight, thinness, and wasting being 25.8, 20.0, 15.8, and 6.8%, respectively. Child’s genotype, (SS), significantly predicted stunting but not underweight.

## Abbreviations

BMIZ: Body Mass Index for age z-score
GHS: Ghana Health Service
HAZ: height for age z-score
PML: Princess Marie Louise Hospital
RDA: Recommended Dietary Allowance
SCD: Sickle cell disease
WAZ: Weight for age z-score
WHZ: Weight for height z-score
